# Sinogram-Affirmed Iterative Reconstruction Negatively Impacts the Risk Category Based on Agatston Score: A Study Combining Coronary Calcium Score Measurement and Coronary CT Angiography

**DOI:** 10.1155/2020/6909130

**Published:** 2020-07-13

**Authors:** Wei Wang, Yan E. Zhao, Li Qi, Chang Sheng Zhou, Meng Jie Lu, Jian Xin Yang, Long Jiang Zhang, Guang Ming Lu

**Affiliations:** ^1^Department of Medical Imaging, Affiliated Jinling Hospital, Medical School of Nanjing University, 305 Zhongshan East Road, Nanjing, Jiangsu 210002, China; ^2^Journal of Medical Postgraduates, Affiliated Jinling Hospital, Medical School of Nanjing University, 305 Zhongshan East Road, Nanjing, Jiangsu 210002, China

## Abstract

**Purpose:**

To assess the impact of sinogram-affirmed iterative reconstruction (SAFIRE) on risk category for coronary artery disease by combining coronary calcium score measurement and coronary CT angiography (CCTA).

**Materials and Methods:**

Eighty-nine patients (64.0% male) older than 18 years (64.4 ± 10.3 years) underwent coronary artery calcium scanning and prospectively ECG-triggered sequential CCTA examination. All raw data acquired in coronary artery calcium scanning were reconstructed by both filtered back projection (FBP) and SAFIRE algorithms with 5 different levels. Objective image quality and calcium quantification were evaluated and compared between FBP and all SAFIRE levels by the Sphericity Assumed test or Greenhouse-Geisser *ε* correction coefficient. Coronary artery stenosis was assessed in CCTA. Risk categories of all patients and of the patients with coronary artery stenosis in CCTA were compared between FBP and all SAFIRE levels by the Friedman test.

**Results:**

The reconstruction protocol from traditional FBP to SAFIRE 5 was associated with a gradual reduction in CT value and image noise (*P* < 0.001) but associated with a gradual improvement in the signal-to-noise ratio (*P* < 0.001). There was a gradual reduction in coronary calcification quantification (Agatston score: from 73.5 in FBP to 38.1 in SAFIRE 5, *P* < 0.001) from traditional FBP to SAFIRE 5. There was a significant difference for the risk category between FBP and all levels of SAFIRE in all patients (from 3.5 in FBP to 3.2 in SAFIRE 5, *P* < 0.001) and in the patients with coronary artery stenosis in CCTA (from 4.0 in FBP to 3.6 in SAFIRE 5, *P* < 0.001).

**Conclusions:**

SAFIRE significantly reduces coronary calcification quantification compared to FBP, resulting in the reduction of risk categories based on the Agatston score. The risk categories of the patients with coronary artery stenosis in CCTA may also decline. Thus, SAFIRE may lead risk categories to underestimate the existence of significant coronary artery stenosis.

## 1. Introduction

Coronary artery calcifications have been regarded as one of the specific performances of atherosclerosis; the scope and amount of deposition of calcium are closely correlated with the degree of coronary atherosclerotic plaque. It has been confirmed that the degree of coronary artery calcification is associated with the risk of adverse cardiac events. Diagnosis and quantification of coronary artery calcifications by cardiac computed tomography (CT) become very important [1–6].

Calcium score measurement based on the Agatston score is a method maturely used to assess the deposition degree of calcium in the coronary artery [7]. The appropriate reconstruction process is a prerequisite of the accurate measurement of coronary calcification. Sinogram-affirmed iterative reconstruction (SAFIRE), an advanced iterative reconstruction (IR) technique instead of the traditional filtered back projection (FBP) approach, has been widely introduced. Previous studies have confirmed that this algorithm can permit preserved image quality while reducing radiation dose in coronary CT angiography (CCTA) with well-established reconstruction kernels [8–15].

In recent years, some studies paid attention to the influence of IR, such as SAFIRE [16–18], adaptive statistical IR (ASIR) [19–21], advanced modeled IR (ADMIRE) [22], hybrid IR (HIR) [23], model-based IR [24], and adaptive iterative dose reduction (AIDR) [25], on accurate measurement of the coronary calcium score. Kurata et al. [16] reported that SAFIRE techniques significantly affected the calcium score measurement, which potentially alters subsequent cardiovascular risk in current medicine practice. Willemink et al. [17] found the similar trend. However, to the best of our knowledge, none of the above studies were combined with CCTA to assess the efficiency of the risk category, which was based on coronary calcium score measurements with SAFIRE reconstruction to evaluate coronary artery stenosis. Thus, the purpose of this study was to assess the impact of SAFIRE on the evaluation ability of the risk category for coronary artery disease by combining coronary calcium score measurement and CCTA.

## 2. Materials and Methods

### 2.1. Study Population

This prospective study was approved by the local institutional review board, and written informed consents were obtained from all patients. From October 2015 to April 2016, ninety-three consecutive patients were collected in this study for clinically known or suspected coronary artery disease such as old myocardial infarction, electrocardiograph (ECG) abnormalities, paroxysmal tachycardia, palpitation, precordial pain or discomfort, and chest distress or pain or for physical examination. The patients' baselines and clinical data were recorded. Inclusion criteria were age greater than 18 years, sinus rhythm, heart rate (HR) variability ≤ 30 beats per minute (bpm), and Agatston score above zero in FBP protocol. Exclusion criteria were as follows: (1) patients who had allergic reactions to iodinated contrast medium before, (2) patients who had acute heart failure or impaired renal function (creatinine level ≥ 120 *μ*mol/L), (3) pregnant women, and (4) patients who had received revascularization including stent implantation or coronary artery bypass graft [26].

### 2.2. CT Acquisition Parameters

All patients received 0.3 mg of nitroglycerin (0.1 mg per dose, Nitroglycerin Inhaler; Jingwei Pharmacy Co, Ltd, Jinan, China) sublingually in order to dilate the coronary arteries at 5 min before CT examination. HR management was not implemented generally for the conventional sequential 120 kVp CCTA [26].

All examinations were performed in a dual-source CT system (Somatom Flash; Siemens Medical Solutions, Forchheim, Germany). All patients first underwent coronary artery calcium scanning, followed by CCTA examination. Both scans were performed in a craniocaudal direction during inspiratory breathhold with prospectively ECG-triggered sequential scanning. The following parameters were the same for both scans: detector collimation, 128 × 0.6 mm; gantry rotation time, 280 ms/rot; tube voltage, 120 kVp. Automated tube current modulation (CARE Dose 4D, Siemens) was used with an effective tube current–time product of 80 mAs per rotation for coronary artery calcium scanning and 390 mAs per rotation for CCTA scanning. A bolus of 60 mL contrast agent (Ultravist 370 mg I/mL; Bayer Healthcare, Berlin, Germany) was injected into the antecubital vein at a rate of 5 mL/s followed by 40 mL of saline solution with the same injection rate. A bolus tracking triggered scanning technique was used to control contrast agent application. A region of interest (ROI) was set in the aortic root; image acquisition was started 4 seconds after the CT attenuation values reached a predefined threshold of 100 Hounsfield units (HU) [26].

### 2.3. CT Image Reconstruction and Analysis

All original data acquired in coronary artery calcium scanning were reconstructed by both FBP and SAFIRE algorithms with 5 levels. Parameters for the FBP reconstruction comprised a noniterative convolution kernel of B30f, 3.0 mm reconstruction slice thickness, 3.0 mm increment, and fast planning field of view (FOV) to include the whole heart. SAFIRE used the I30f convolution kernel, which has a modulation transfer function similar to B30f used in FBP. Reconstruction slice thickness, reconstruction increment, and FOV for SAFIRE were kept identical to those for the FBP reconstructions. In SAFIRE, specific settings, which were chosen according to the manufacturer's recommendation, were two iterations in raw data and five iterations in the image domain. Algorithm strength was from level 1 to level 5 (SAFIRE levels 1-5).

All reconstructed images were transferred to a dedicated workstation (3D Workplace, Siemens). The nonenhanced CT data sets for the coronary calcium score were evaluated using semiautomatic software (Syngo Calcium Scoring CT, Siemens, Germany) to obtain the Agatston score. All distributions of pixels with a density above a defined threshold (130 HU) were identified and colour marked automatically. Coronary artery calcium scores were separately calculated for each coronary artery: left main coronary artery (LMA), left anterior descending artery (LAD), left circumflex artery (LCX), and right coronary artery (RCA). Lesions were selected and assigned into the above four coronary arteries manually by one observer (W.W. with 3 years' experience in CCTA). The lesions in the branches of the above coronary arteries were classified as the main arteries. The lesion at the junction of the two main arteries was distributed according to the location of its center. From the selected areas, the software automatically calculated the lesion number, volume (mm^3^), equivalent mass (mg), and Agatston score. The total coronary calcium score was summed and recorded. The CT attenuation value of the main (largest volume) calcium lesion and standard deviation (SD) of CT attenuation of the aortic root was measured for each subject. The circular ROI as large as possible was placed in the center of the main (largest volume) calcium lesion and aortic root lumen to measure their CT value or SD. For all measurements in FBP and SAFIRE 1-5 images in each subject, we selected the same calcium lesions at the same slice; the ROI size was kept the same to reduce measurement bias as possible. The SD of CT attenuation of the aortic root was defined as image noise. The signal-to-noise ratio (SNR) was calculated according to the following equations: SNR = CT attenuation value of the main calcium lesion/image noise [27].

CCTA image reconstruction was performed with a section thickness of 0.75 mm, an increment of 0.5 mm, SAFIRE algorithms at strength 3 [28], and a medium soft-tissue convolution kernel (I26f). All reconstructed images were transferred to the same dedicated workstation (3D Workplace, Siemens) equipped with cardiac postprocessing software (Syngo.Via CT Coronary, Siemens). Image postprocessing techniques included curved multiplanar reformation and volume rendered reformation. Coronary artery stenosis was assessed in CCTA. The coronary artery was divided into 15 segments according to the coronary artery segmental model formulated by the American Heart Association [29]. RCA included segments 1 to 4, LMA was segment 5, LAD included segments 6 to 10 (ramus medianus was assigned as segment 9 just like the first diagonal branch), and LCX included segments 11 to 15. Two cardiovascular radiologists (Y.E.Z. and W.W. with 8 and 3 years' experience in reading CCTA, respectively) who were blinded to the results of the Agatston score independently determined the presence or absence of stenosis on the basis of per-segment. Unassessable segments such as significant motion artifact or noise, discontinuous or unclear vessel walls, massive coronary calcification, or insufficient image contrast were excluded. All patients' information was anonymous during image analysis. When there was a controversy about the results, both radiologists achieved consensus through consulting to determine the final result in a joint assessment. Only ≥50% diameter stenosis was classified as significant. The artery was defined as a stenotic coronary artery if a stenosis appeared in at least one segment.

### 2.4. Risk Category Evaluation

To evaluate cardiovascular risk of coronary artery disease, all patients were distributed in one risk category based on their absolute Agatston scores. The risk category levels 1–5 correspond to Agatston score = 0, 0 < Agatston score ≤ 10, 10 < Agatston score ≤ 100, 100 < Agatston score ≤ 400, and Agatston score > 400, respectively [16, 18, 19, 30, 31]. Considering the changes of Agatston scores caused by different reconstruction protocols, the changes in patient risk categories between FBP and all SAFIRE levels were also evaluated for all patients and for the patients with at least one stenotic coronary artery in CCTA.

### 2.5. Statistical Analysis

Data were analyzed by using the SPSS 16.0 software package (SPSS Inc., Chicago, IL, USA). Quantitative data were expressed as mean values ± SD for normal distributions and median (interquartile range) for nonnormal distributions; categorical data were expressed as frequencies or percentages. The comparisons on the CT attenuation value of the main calcium lesion, image noise, SNR, lesion number, volume, equivalent mass, and Agatston score between FBP and all SAFIRE levels were performed by using the Sphericity Assumed test or Greenhouse-Geisser *ε* correction coefficient. The comparison on reduction of the Agatston score from FBP between patients with low values and high values of coronary calcifications was performed by using the independent-samples *t*-test for normal distributions and Mann-Whitney *U* test for nonnormal distributions. The comparison on risk categories between FBP and all SAFIRE levels was analyzed by the Friedman test. *P* values of less than 0.05 were considered as significant. The post hoc analysis after the Friedman test was performed by the Wilcoxon one-sample test with the Bonferroni method (*P* values of less than 0.05/15 were considered as significant).

## 3. Results

### 3.1. Study Population

A total of eighty-nine patients were included in the effective analysis. Four patients were ultimately excluded, including one patient who had received coronary artery bypass graft and three patients with an Agatston score equal to zero in FBP protocol. The patients' demographic and clinical characteristics, including sex, age, height, body weight, body mass index, scan HR, cardiovascular risk factors, chest symptom, and radiation dose including volumetric CT dose index (CTDI_vol_), dose-length product (DLP), and effective dose (ED), are shown in Table 1.

### 3.2. Objective Assessment of Image Quality

The CT attenuation value of the main (largest volume) calcium lesion, image noise, and SNR are shown in Table 2. There were significant differences for CT value, image noise, and SNR between FBP and all levels of SAFIRE (all *P* < 0.001). The reconstruction protocol from traditional FBP to SAFIRE 5 was associated with a gradual reduction in the CT value and image noise (*P* < 0.001) but associated with a gradual improvement in SNR (*P* < 0.001).

### 3.3. Calcium Scoring

**Figure 1:**
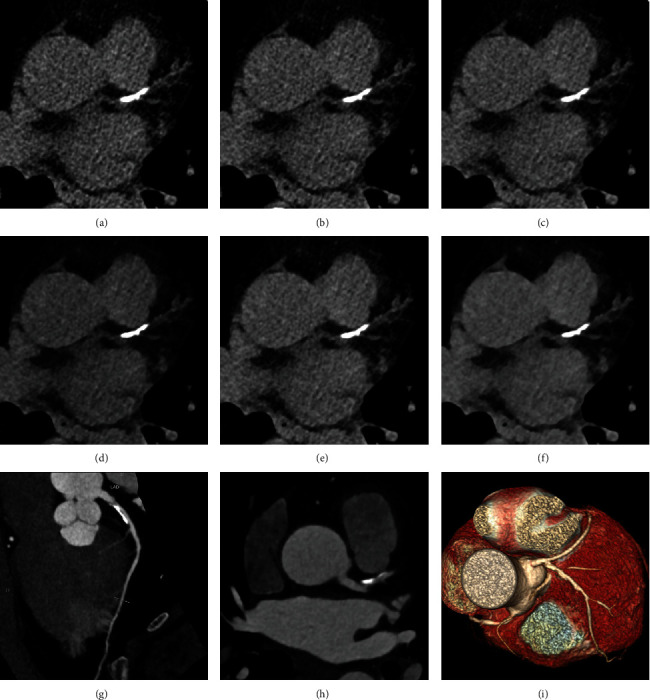
An example in a 71-year-old woman with suspected coronary artery disease. (a–f) Transverse CT images reconstructed with FBP (a), SAFIRE 1 (b), SAFIRE 2 (c), SAFIRE 3 (d), SAFIRE 4 (e), and SAFIRE 5 (f) and 3.0 mm reconstruction slice thickness. A calcified plaque located in the proximal segment of the left anterior descending artery is clearly shown in FBP and SAFIRE 1-5 images with the total Agatston score of 179.3, 163.9, 157.8, 154.2, 145.4, and 134.9, respectively. (g–i) Curved planar reformation of the left anterior descending artery (g), transverse CT image (h), and volume rendered reformatted image (i) in CCTA. A moderate stenosis caused by this calcified plaque is located in the proximal segment of the left anterior descending artery.

Data acquired included the lesion number, volume, equivalent mass, and Agatston score (Table 2). There were significant differences for the lesion number, calcium volume, equivalent mass, and Agatston score between FBP and all levels of SAFIRE (all *P* < 0.001). The reconstruction protocol from traditional FBP to SAFIRE 5 was associated with a gradual reduction in the lesion number, calcium volume, equivalent mass, and Agatston score (*P* < 0.001) (Figure 1). Compared with FBP, calcium volumes were reduced by 15.7 ± 8.0%, 23.7 ± 11.8%, 30.1 ± 14.3%, 37.6 ± 15.9%, and 44.1 ± 16.7% in SAFIRE levels 1, 2, 3, 4, and 5, respectively. In comparison with FBP, equivalent mass was reduced by 14.8 ± 8.1%, 22.5 ± 11.0%, 28.9 ± 13.6%, 35.4 ± 14.6%, and 42.0 ± 16.0% in SAFIRE levels 1, 2, 3, 4, and 5, respectively. In comparison with FBP, there was a decrease of 15.7 ± 11.5%, 23.3 ± 12.9%, 30.6 ± 15.0%, 37.7 ± 15.4%, and 44.7 ± 16.5% in the Agatston score for SAFIRE strength levels 1, 2, 3, 4, and 5, respectively. Thus, as the strength of SAFIRE increased, the calcium score was decreased. Moreover, the Agatston score reduction compared with FBP was more marked in patients with a low coronary calcification score (Agatston score < 100) than patients with a high coronary calcification score (Agatston score > 100) in SAFIRE 1 (17.7 ± 14.5% vs. 12.8 ± 3.7%, *P* = 0.024), SAFIRE 2 (26.8 ± 15.4% vs. 18.5 ± 5.5%, *P* = 0.001), SAFIRE 3 (35.4 ± 16.6% vs. 22.8 ± 6.7%, *P* < 0.001), SAFIRE 4 (42.6 ± 16.5% vs. 29.0 ± 7.7%, *P* < 0.001), and SAFIRE 5 (50.4 ± 17.1% vs. 33.9 ± 8.0%, *P* < 0.001).

The Agatston score of one patient (1.1%), which was evaluated as 1.5, 0.9, 0.5, and 0.1 by using FBP and SAFIRE 1-3, respectively, decreased to zero using SAFIRE 4 and 5. In three patients (3.4%), Agatston scores were increased using SAFIRE compared with FBP. Among them, the Agatston score in the first patient was increased by 31.2%, 9.1%, and 1.3% in SAFIRE 1, 2, and 3, respectively; the Agatston score in the second patient was increased by 24.2% and 12.2% in SAFIRE 1 and 2, respectively; and the Agatston score in the third patient was increased by 4.8% in SAFIRE 1. In addition, one patient (1.1%) has the same Agatston score to FBP by using SAFIRE 1.

### 3.4. Risk Category of all Patients

The variability of the Agatston score caused by different SAFIRE reconstruction protocols resulted in different risk categories. There was significant difference for the risk category between FBP and all levels of SAFIRE (*P* < 0.001). From FBP to SAFIRE 5, the mean risk category levels were 3.5 ± 0.9, 3.4 ± 0.9, 3.4 ± 0.9, 3.3 ± 0.9, 3.2 ± 0.9, and 3.2 ± 0.9, respectively. From FBP to SAFIRE 5, the percentages of patients were gradually increased for the low risk category (e.g., 0 < Agatston score ≤ 10) and decreased for the high risk category (e.g., Agatston score > 400) (Table 3). In total, 24 (27.0%) patients had to be reassigned to lower risk categories from the previous protocols: 7 patients in SAFIRE 1 (risk category level: three patients from 5 to 4, one patient from 4 to 3, and three patients from 3 to 2), 1 patient in SAFIRE 2 (risk category level: from 5 to 4), 5 patients in SAFIRE 3 (risk category level: three patients from 4 to 3 and two patients from 3 to 2), 9 patients in SAFIRE 4 (risk category level: two patients from 5 to 4, two patients from 4 to 3, four patients from 3 to 2, and one patient from 2 to 1 whose Agatston score decreased to zero) (Table 3), and 2 patients in SAFIRE 5 (risk category level: from 5 to 4 and from 4 to 3).

### 3.5. Risk Category of Patients with Coronary Artery Stenosis in CCTA

**Figure 2:**
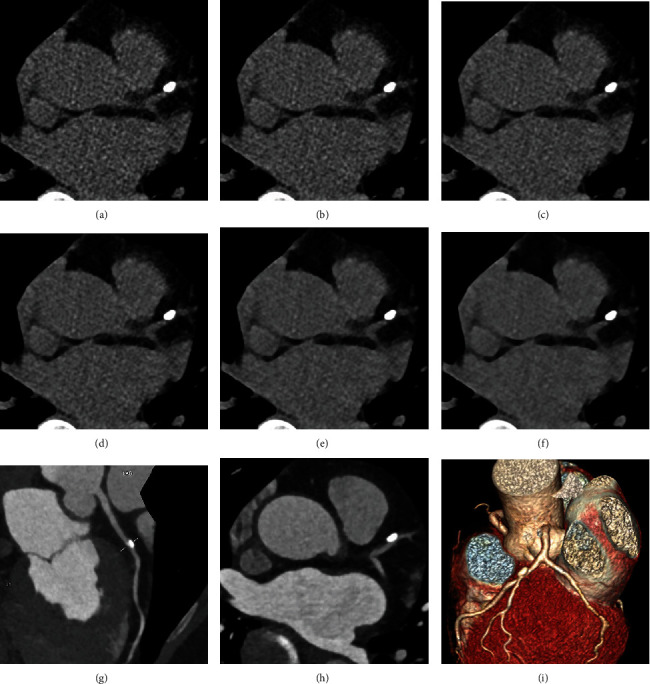
An example in a 61-year-old woman with suspected coronary artery disease. (a–f) Transverse CT images reconstructed with FBP (a), SAFIRE 1 (b), SAFIRE 2 (c), SAFIRE 3 (d), SAFIRE 4 (e), and SAFIRE 5 (f) and 3.0 mm reconstruction slice thickness. A calcified plaque located in the proximal segment of the left anterior descending artery is clearly shown in FBP and SAFIRE 1-5 images with the total Agatston score of 124.6, 104.3, 100.9, 92.6, 86.3, and 80.3, respectively. (g–i) Curved planar reformation of the left anterior descending artery (g), transverse CT image (h), and volume rendered reformatted image (i) in CCTA. A moderate stenosis caused by this calcified plaque is located in the proximal segment of the left anterior descending artery.

CCTA from all patients showed that 79 arteries in 49 cases had significant stenosis in at least one coronary artery segment, including 29 patients with 1 stenotic coronary artery and 20 patients with ≥2 stenotic coronary arteries (Figure 1). There was a significant difference for the risk category of patients with at least one stenotic coronary artery between FBP and all levels of SAFIRE (*P* < 0.001), including patients with 1 stenotic coronary artery (*P* < 0.001) and patients with ≥2 stenotic coronary arteries (*P* = 0.001). From FBP to SAFIRE 5, the mean risk category levels were 4.0 ± 0.7, 3.9 ± 0.7, 3.9 ± 0.7, 3.8 ± 0.8, 3.7 ± 0.8, and 3.6 ± 0.8, respectively. The percentages were gradually increased for the low risk category (e.g., 0 < Agatston score ≤ 10 and 10 < Agatston score ≤ 100) but decreased for the high risk category (e.g., Agatston score > 400) from FBP to SAFIRE 5 (Table 4). In addition, the prevalence of coronary artery stenosis was gradually increased for the low risk category (e.g., 0 < Agatston score ≤ 10 and 10 < Agatston score ≤ 100) from FBP to SAFIRE 5 (Table 4). In total, 18 cases who belonged to the above 24 were redistributed to lower risk categories from the previous scheme: 5 patients in SAFIRE 1 (risk category level: three patients from 5 to 4, one patient from 4 to 3, and one patient from 3 to 2), 1 patient in SAFIRE 2 (risk category level: from 5 to 4), 4 patients in SAFIRE 3 (risk category level: three patients from 4 to 3 and one patient from 3 to 2) (Figure 2), 6 patients in SAFIRE 4 (risk category level: two patients from 5 to 4, two patients from 4 to 3, and two patients from 3 to 2), and 2 patients in SAFIRE 5 (risk category level: from 5 to 4 and from 4 to 3).

## 4. Discussion

Different from the previous studies, this is the first research to assess the impact of SAFIRE on the evaluation efficiency of the risk category for coronary artery disease by combining coronary calcium score measurement and CCTA.

This study demonstrates that SAFIRE can significantly reduce the quantification of coronary calcification including the lesion number, calcium volume, equivalent mass, and Agatston score compared to traditional FBP. Gebhard et al. [20] reported that compared to FBP, the volume (reduction from 3.7% to 18.6%) and Agatston scores (reduction from 6.0% to 22.4%) were significantly reduced when increasing the ASIR algorithm from 20% to 100%, while they found no effects on the mass. van Osch et al. [19] also found a small reduction in mass scores when using ASIR. The explanation from Gebhard et al. for the nonsignificant effect of ASIR on the mass score was that the mass score might have a lower susceptibility to partial volume effects. In contrast to the above observations using ASIR, Kurata et al. [16] reported that the mass decreased similarly to the volume and Agatston score using SAFIRE, and reduction in the Agatston score, volume, and mass was 48%, 48%, and 45%, respectively, by using the maximum of the amount of SAFIRE. Similar to the previous study [16], we also found that the reductions in the Agatston score, mass, and volume of SAFIRE compared to FBP were larger than the reductions with ASIR. Using the maximum strength of SAFIRE, the reduction in calcium volume, equivalent mass, and Agatston score was 44.1%, 42.0%, and 44.7%, respectively. This may be explained by the discrepancies among the IR algorithms. SAFIRE takes advantage of not only raw data but also image space data. ASIR is primarily based on the raw data with the mixture of noise-free images and FBP images to restore a more familiar image quality [32]. Moreover, the differences of study population may also result in these discrepancies of reductions in the calcium scores. Patients of Gebhard et al. had a higher median Agatston score of 345, but the patient group in this study had a relatively lower median Agatston score in FBP of 73.5. The reduction of the Agatston score caused by IR is more apparent in patients with a low Agatston score.

In addition, we found that Agatston scores in three patients were increased, and this indicator in another patient was the same using SAFIRE 1 compared with FBP. The underlying mechanisms of different effects of SAFIRE on the Agatston score including reduction, increase, and maintenance are complex. The most influencing factors, for instance, lesion number, calcium volume, equivalent mass, scan HR, body weight, body mass index, modulated tube current, and associated image noise [16], will be evaluated in the following study.

We used absolute Agatston scores to determine risk categories in this study. Between FBP and all levels of SAFIRE, the risk category was changed significantly, and 24 patients had been reassigned to lower risk categories, which was similar to 22 patients of Kurata et al.'s study with the same IR algorithm. In addition, our shift of 27% was obviously higher than Gebhard et al.'s 18% shift, but relatively approximate to van Osch et al.'s shift of 29%, both of which used ASIR, probably because we used the same criterion to determine the risk category as van Osch et al. and the median Agatston score of our patient group was close to van Osch et al.'s median Agatston score of 81. This change of risk category may consequently impact clinical decision-making on follow-up and treatment strategies and should be taken into account when using SAFIRE.

Although we have cited some previous studies about the impact of IR algorithms on the Agatston score-based risk category, we were not sure whether this effect extended to evaluation efficiency of the risk category for coronary artery disease. A few studies with a combination of Agatston score and CCTA [33, 34] have shown that coronary calcium scoring provided useful information in the diagnosis of coronary artery stenosis. It has been reported that the prevalence of significant coronary artery stenoses increased with increasing calcium amounts and extensive calcium may be associated with a high prevalence of significant coronary artery stenoses [34]. In this study, we also found this tendency in FBP. The risk categories of the patients with coronary artery stenosis in CCTA were all greater than or equal to 3 (10 < Agatston score ≤ 100). The prevalence of significant coronary artery stenosis increased with increasing risk categories, which was up to 100% on the highest category. But this tendency has changed with strengthening the SAFIRE algorithm. The percentages of patients and the prevalence of coronary artery stenosis were both gradually increased for the low risk category. Although the prevalence in risk category 5 (Agatston score > 400) was still 100%, the percentages gradually decreased. It could be seen that some patients with coronary artery stenosis were falsely redistributed to the lower risk category, the evaluation efficiency of which was negatively influenced. Consequently, the decline in risk categories caused by SAFIRE may lead to underestimating the existence of significant coronary artery stenosis.

Moreover, even though SAFIRE can reduce image noise and improve image quality, the result of this study found that the effect on risk category of this technique seemed to be more pronounced in patients with coronary artery stenosis (total number of the patients redistributed to lower risk categories: 18 cases in the patients with coronary artery stenosis vs. 24 cases in all patients). Even in the lowest strength level (SAFIRE 1), the risk categories of 5 patients with coronary artery stenosis have falsely declined. There have been some changes in the percentages and the prevalence of this strength level relative to FBP. This negative influence was accumulating with strengthening SAFIRE. More specifically, for each additional strength level, more patients with coronary artery stenosis were incorrectly reassigned. As a result, the risk categories have significantly decreased compared with FBP or lower strength levels. Thus, based on our observation in this study, any strength level of SAFIRE was not recommended preferentially for clinical routine coronary calcium quantification. However, further work is still deserved to clarify the more detailed role of SAFIRE on coronary calcium quantification.

Some limitations in our study deserve further consideration. First, our sample size was relatively small, especially for the prevalence of coronary artery stenosis. Second, the median Agatston score in our study was 73.5, indicating that the studied patients were a low-risk population which potentially limits the generalization of our results. Third, we only studied the effect of a specific IR algorithm (SAFIRE) on a specific CT scanner to minimize the potential bias, but different reconstruction algorithms and different scanners may introduce much more variability of coronary calcium quantification. Fourth, there were only a few unassessable segments (vessels) in this study, which were not recorded when the presence or absence of stenosis was being determined. Last, this study did not address the influence of the coronary calcium score on the diagnostic accuracy of CCTA because no invasive coronary angiography was performed in all patients. We assumed that the diagnostic results of CCTA were accurate.

In conclusion, SAFIRE significantly reduces coronary calcification quantification compared to FBP, resulting in the reduction of risk categories based on the coronary calcium score. The risk categories of the patients with coronary artery stenosis in CCTA may also decline. Thus, SAFIRE may lead risk categories to underestimate the existence of significant coronary artery stenosis.

## Figures and Tables

**Table 1:** Patient demographic, clinical, and CT acquisition characteristics.

Variables	Study population
Sex (*n*)	57/32 (M/F)
Age (years)	64.4 ± 10.3
Height (m)	1.65 ± 0.08
Body weight (kg)	67.5 ± 11.8
Body mass index (kg/m^2^)	24.4 ± 4.4
Scan HR (bpm)	
Coronary artery calcium scanning	74.5 (65.0-84.8)
CCTA examination	74.6 ± 14.7
Cardiovascular risk factors	
Hypertension	44 (49.4%)
Hypercholesterolemia	15 (16.9%)
Diabetes mellitus	13 (14.6%)
Smoking	17 (19.1%)
Family history of CAD	1 (1.1%)
Chest symptoms	
Atrial premature beats	4 (4.5%)
Atrial flutter	1 (1.1%)
Atrial fibrillation	4 (4.5%)
Other ECG abnormalities	10 (11.2%)
Paroxysmal tachycardia	4 (4.5%)
Palpitation	6 (6.7%)
Precordial pain or discomfort	9 (10.1%)
Chest distress or pain	33 (37.1%)
Coronary artery calcium scanning	
CTDIvol (mGy)	2.5 ± 0.7
DLP (mGy∗cm)	31.0 (26.0-40.0)
ED (mSv)	0.5 ± 0.1
CCTA examination	
CTDIvol (mGy)	31.0 (24.4-42.9)
DLP (mGy∗cm)	366.0 (261.0-501.0)
ED (mSv)	5.1 (3.7-7.0)

HR: heart rate; bpm: beats per minute; CCTA: coronary CT angiography; CAD: coronary artery disease; ECG: electrocardiograph; CTDI_vol_: volumetric CT dose index; DLP: dose-length product; ED: effective dose.

**Table 2:** The lesion number, volume, equivalent mass, Agatston score, CT attenuation value of the main calcium lesion, image noise, and SNR in FBP and all levels of SAFIRE.

Index	FBP	SAFIRE 1	SAFIRE 2	SAFIRE 3	SAFIRE 4	SAFIRE 5	*P* value
Lesion number (*n*)	5.0 (2.0-7.0)	4.0 (2.0-7.0)	4.0 (1.0-6.0)	4.0 (1.0-5.0)	3.0 (1.0-5.0)	3.0 (1.0-5.0)	<0.001
Volume (mm^3^)	54.6 (20.5-164.7)	48.9 (16.6-141.5)	40.7 (14.9-135.2)	36.2 (13.5-129.1)	33.4 (11.7-119.6)	31.0 (10.3-111.2)	<0.001
Equivalent mass (mg)	12.1 (4.7-38.5)	10.5 (4.4-35.2)	9.6 (3.8-34.6)	8.9 (3.4-33.7)	8.2 (2.8-30.8)	7.3 (2.3-28.3)	<0.001
Agatston score	73.5 (28.9-198.3)	63.5 (20.1-172.0)	57.7 (18.2-164.8)	51.1 (16.5-159.1)	46.1 (14.4-148.5)	38.1 (12.1-134.9)	<0.001
Lesion CT value (HU)	462.0 ± 208.0	456.5 ± 213.8	445.5 ± 209.7	439.7 ± 207.5	432.4 ± 215.2	425.6 ± 214.7	<0.001
Image noise (HU)	20.7 ± 3.0	18.4 ± 2.7	16.3 ± 2.5	14.1 ± 2.1	12.0 ± 1.8	9.9 ± 1.5	<0.001
SNR	19.8 (14.1-28.6)	22.9 (16.1-31.4)	25.8 (17.0-36.0)	29.1 (19.3-41.4)	33.0 (21.7-47.0)	39.5 (26.6-57.2)	<0.001

FBP: filtered back projection; SAFIRE: sinogram-affirmed iterative reconstruction; SNR: signal-to-noise ratio.

**Table 3:** Patients' number (n1) in different risk categories of all patients (*n* = 89) between FBP and all levels of SAFIRE.

Risk category	FBP	SAFIRE 1	SAFIRE 2	SAFIRE 3^★^	SAFIRE 4^★◎■^	SAFIRE 5^★◎■◆^
Agatston score = 0^#^	0	0	0	0	1 (1.1%)	1 (1.1%)
0 < Agatston score ≤ 10^∗^	11 (12.4%)	14 (15.7%)	14 (15.7%)	16 (18.0%)	19 (21.3%)	19 (21.3%)
10 < Agatston score ≤ 100	40 (44.9%)	38 (42.7%)	38 (42.7%)	39 (43.8%)	37 (41.6%)	38 (42.7%)
100 < Agatston score ≤ 400	25 (28.1%)	27 (30.3%)	28 (31.5%)	25 (28.1%)	25 (28.1%)	25 (28.1%)
Agatston score > 400^∗^	13 (14.6%)	10 (11.2%)	9 (10.1%)	9 (10.1%)	7 (7.9%)	6 (6.7%)

FBP: filtered back projection; SAFIRE: sinogram-affirmed iterative reconstruction.^#^The risk category of one patient was reassigned from 2 to 1 according to the Agatston score reduced to zero using SAFIRE 4. ^∗^The percentages (n1/89) were gradually increased for the low risk category (e.g., 0 < Agatston score ≤ 10) and decreased for the high risk category (e.g., Agatston score > 400).^★^*P* < 0.003 compared with FBP, ^◎^*P* < 0.003 compared with SAFIRE 1, ^■^*P* < 0.003 compared with SAFIRE 2, and ^◆^*P* < 0.003 compared with SAFIRE 3.

**Table 4:** Patients' number (n2) in different risk categories of patients with coronary artery stenosis (*n* = 49) between FBP and all levels of SAFIRE.

Risk category	FBP	SAFIRE 1	SAFIRE 2	SAFIRE 3^★^	SAFIRE 4^★◎■^	SAFIRE 5^★◎■^
Agatston score = 0^#^						
Patient	0	0	0	0	0	0
1 vessel	0	0	0	0	0	0
≥2 vessels	0	0	0	0	0	0
Prevalence	0	0	0	0	0	0
0 < Agatston score ≤ 10^∗^^▲^						
Patient	0	1 (2.0%)	1 (2.0%)	2 (4.1%)	4 (8.2%)	4 (8.2%)
1 vessel	0	1	1	2	3	3
≥2 vessels	0	0	0	0	1	1
Prevalence	0	7.1%	7.1%	12.5%	21.1%	21.1%
10 < Agatston score ≤ 100^∗^^▲^						
Patient	13 (26.5%)	13 (26.5%)	13 (26.5%)	15 (30.6%)	15 (30.6%)	16 (32.7%)
1 vessel	10	10	10	11	11	12
≥2 vessels	3	3	3	4	4	4
Prevalence	32.5%	34.2%	34.2%	38.5%	40.5%	42.1%
100 < Agatston score ≤ 400						
Patient	23 (46.9%)	25 (51.0%)	26 (53.1%)	23 (46.9%)	23 (46.9%)	23 (46.9%)
1 vessel	15	15	15	13	14	13
≥2 vessels	8	10	11	10	9	10
Prevalence	92.0%	92.6%	92.9%	92.0%	92.0%	92.0%
Agatston score > 400^∗^						
Patient	13 (26.5%)	10 (20.4%)	9 (18.4%)	9 (18.4%)	7 (14.3%)	6 (12.2%)
1 vessel	4	3	3	3	1	1
≥2 vessels	9	7	6	6	6	5
Prevalence	100%	100%	100%	100%	100%	100%

FBP: filtered back projection; SAFIRE: sinogram-affirmed iterative reconstruction.^#^The only patient whose Agatston score decreased to zero using SAFIRE 4 had no significant stenosis. ^∗^The percentages (n2/49) were gradually increased for the low risk category (e.g., 0 < Agatston score ≤ 10 and 10 < Agatston score ≤ 100) but decreased for the high risk category (e.g., Agatston score > 400) from FBP to SAFIRE 5. ^▲^The prevalence (n2/n1) of coronary artery stenosis was gradually increased for the low risk category (e.g., 0 < Agatston score ≤ 10 and 10 < Agatston score ≤ 100) from FBP to SAFIRE 5. ^★^*P* < 0.003 compared with FBP; ^◎^*P* < 0.003 compared with SAFIRE 1; ^■^*P* < 0.003 compared with SAFIRE 2.

## Data Availability

The (Excel) data used to support the findings of this study currently need to be kept confidential. Requests for data, (6/12 months) after publication of this article, will be considered by the corresponding author.
